# Composition and Antioxidant Activity of Geopropolis Collected by *Melipona subnitida* (Jandaíra) Bees

**DOI:** 10.1155/2013/801383

**Published:** 2013-07-14

**Authors:** Silvana Alves de Souza, Celso Amorim Camara, Eva Monica Sarmento da Silva, Tania Maria Sarmento Silva

**Affiliations:** ^1^Laboratório de Bioprospecção Fitoquímica, Departamento de Ciências Moleculares, Universidade Federal Rural de Pernambuco, 52171-900 Recife, PE, Brazil; ^2^Colegiado de Zootecnia, Universidade Federal do Vale de São Francisco, 56300-990 Petrolina, PE, Brazil

## Abstract

An investigation of the geopropolis collected by *Melipona subnitida* (jandaíra) stingless bee led to the isolation and characterization of two phenylpropanoids, 6-*O-p*-coumaroyl-*D*-galactopyranose (**1**) and 6-*O*-cinnamoyl-1-*O-p*-coumaroyl-**β**-*D*-glucopyranose (**2**), and seven flavonoids, 7-*O*-methyl-naringenin (**3**), 7-*O*-methyl aromadendrin (**4**), 7,4′-di-*O*-methyl aromadendrin (**5**), 4′-*O*-methyl kaempferol (**6**), 3-*O*-methyl quercetin (**7**), 5-*O*-methyl aromadendrin (**8**), and 5-*O*-methyl kaempferol (**9**). The structure of the new phenylpropanoid (**1**) was established from IR, LC-ESI-MS, and NMR spectral data, including 2D NMR experiments. The extract and fractions demonstrated significant antioxidant activity in DPPH, ABTS, and **β**-carotene/linoleic acid tests.

## 1. Introduction

 Geopropolis is a special type of propolis, or bee glue, prepared by stingless bees (Meliponinae). As presented [1], geopropolis is a mixture of plant resins and waxes and earth. Honeybees (*Apis*) do not use soil material when preparing traditional propolis [2]. Previous investigations on tropical propolis have concentrated almost exclusively on *Apis mellifera *bee glue. In tropical South America, there are indigenous stingless bee species (Meliponinae) that collect geopropolis, such as the *Melipona subnitida* Ducke “jandaíra” bees native to northeastern Brazil. The primary importance of this species is associated with environmental conservation and fruit production, as they pollinate wild plants and cultivated crops in the semiarid Caatinga (shrub vegetation) and humid pre-Amazonian forest regions [3].

 The chemical composition of geopropolis or propolis depends on the specificity of the local flora at the collection site. Many constituents have been identified principally from propolis, and phenolic compounds, such as flavonoids, phenolic acids, and phenolic acid esters, have been reported as major constituents of propolis from tropical zones [4].

 An investigation of the phenolic constituents of propolis and geopropolis from Venezuela reported the presence of prenylated benzophenones in all the samples [5], in 1993. The chemical compositions of the propolis of five indigenous bee species along with the honeybee did not differ. Bankova et al. [6] identified over 50 substances, mainly phenolic compounds, in Brazilian geopropolis from *Melipona compressipes*, *Melipona quadrifasciata* anthidioides, and *Tetragona clavipes*. Twenty-one samples of Brazilian geopropolis from 12 different species of stingless bees were analyzed, and the presence of such compounds as di- and triterpenes and gallic acid was detected. The same samples showed activity against *Staphylococcus aureus* and cytotoxic activity [7]. Investigations of geopropolis from *Melipona fasciculata *showed antimicrobial activity against *S. mutans* and* C. albicans *as well as an immunomodulatory action due to the increase in anti-inflammatory cytokines [8].

 Propolis is a powerful antioxidant. An antioxidant is a molecule capable of slowing or preventing the oxidation of other molecules. The radical theory in human physiology claims that active free radicals are involved in almost all cellular degradation processes and lead to cell death. Oxidative stress is thought to contribute to the development of chronic and degenerative diseases, such as cancer, autoimmune disorders, aging, cataract, rheumatoid arthritis, and cardiovascular and neurodegenerative diseases [9]. The antioxidant property of propolis is due to its high concentration of phenolics and other antioxidant compounds [10]. Propolis or geopropolis is a potential supplement for preventing chronic degeneration diseases.

 As a part of our continuing study of the chemical and antioxidant activity of *Apis* and *Melipona* products [3, 11–13], this study undertook the isolation of a new phenylpropanoid galactose ester from the geopropolis of jandaíra (*Melipona subnitida*) in addition to eight phenols. This paper describes the isolation and characterization of the new compound and the antioxidant activity of this geopropolis.

## 2. Experimental

### 2.1. General

Melting points were determined using a Kofler hot stage and are uncorrected. The infrared absorption spectra were recorded in KBr pellets using a Varian 640 FT-IR spectrophotometer with a PIKE ATR accessory operating in the 4000–400 cm^−1^ range. The LC-ESI-MS was obtained in positive electrospray mode using an Esquire 3000 Plus instrument (Bruker). TLC plates were run using 60 F_254_ silica gel (Merck). Sephadex LH-20 (Sigma) was employed for gel permeation chromatography. ^1^H and ^13^C NMR spectra were obtained using a Bruker DRX 500 (500 MHz for ^1^H; 125 MHz for ^13^C) and Bruker DPX300 (300 MHz for ^1^H; 75 MHz for ^13^C) in DMSO-*d*_6_. The optical rotation was determined using a KRUESS Optronic spectrometer. All solvents used were of commercial HPLC grade.

### 2.2. Geopropolis Sample

The *M. subnitida* “jandaíra” geopropolis sample was collected in January 2010 at Sítio Riacho, which is in the municipality of Vieirópolis, a semiarid region in the state of Paraiba, Brazil.

### 2.3. Preparation of  Extract and Fractions

Geopropolis (550 g) was extracted with ethanol in an ultrasonic water bath. The combined ethanolic extract was completely evaporated under reduced pressure to afford a brown residue (25.8 g). A portion of the ethanolic extract (15.7 g) was suspended in MeOH:H_2_O and partitioned with hexane and ethyl acetate to yield the corresponding soluble fractions, yielding hexane (4.8 g), ethyl acetate (4.5 g), and MeOH:H_2_O (0.7 g) fractions. The ethyl acetate fraction that showed the most significant antioxidant activity was used for the further fractionation and isolation of individual compounds.

### 2.4. Isolation and Identification of Compounds

A portion of the EtOAc fraction (4.5 g) was subjected to chromatography on a Sephadex LH-20 column with methanol as the mobile phase. Compounds **1** (18.5 mg), **2** (8.5 mg), **3** (2.5 mg), **4** (7.0 mg), **5** (5.5 mg), **6** (2.8 mg), and **7** (9.3 mg) and the mixture of **8** and **9** (4.5 mg) were purified by semipreparative HPLC on a Luna Phenomenex RP-18 column (21 mm × 250 mm × 5 *μ*m) and detected at 320 nm at a flow rate of 15 mL/min using a mobile phase of H_2_O (A) and methanol (B) as follows: 0–5 min, 70–80% B; 5–10 min, 80–90% B; 10–15 min, 90–100% B; and 15-16 min, 100% B. The purity of the compounds was assessed via analytical HPLC with diode array detection. The structures of all the isolated compounds were elucidated based on ^1^H-NMR, ^13^C-NMR, MS, IR, and UV data.

6-*O*-*p*-Coumaroyl-*D*-galactopyranose (**1**). Amorphous white powder. MP: 165–167°C [*α*]_D_ = +26.7 (MeOH, c 0.35, 25°C), LC-ESI-MS (positive mode) *m/z* 327 [M + H]^+^, *m/z* 349 [M + Na]^+^, *m*/*z* 309 [M + H−H_2_O]^+^, *m*/*z* 165 [M−galactose]^+^. UV *λ*_max⁡_ = 310 nm. IR (KBr) *ν*_max⁡_ : 3400, 1690 (C=O), 1607, 1513 (C=C from aromatic rings). ^1^H NMR (DMSO-*d*_6_, 300 MHz; see Table 1), ^13^C NMR (DMSO-*d*_6_, 125 MHz; see Table 1).

 6-*O*-Cinnamoyl-1-*O*-*p*-coumaroyl-*β*-*D*-glucopyranose (**2**). LC-ESI-MS *m*/*z* 455 [M−H]^+^, UV *λ*_max⁡_ = 310 nm.
 7-*O*-Methyl-naringenin (**3**). LC-ESI-MS *m*/*z* 287 [M + H]^+^, UV *λ*_max⁡_ = 286.
 7-*O*-Methyl aromadendrin (**4**). LC-ESI-MS *m*/*z* 303 [M + H]^+^, UV *λ*_max⁡_ = 290.
 7,4′-Di-*O*-methyl aromadendrin (**5**). LC-ESI-MS *m*/*z* 317 [M + H]^+^, UV *λ*_max⁡_ = 288.
 4′-*O*-Methyl kaempferol (**6**). LC-ESI-MS *m*/*z* 301 [M + H]^+^, UV *λ*_max⁡_ = 292.
 3-*O*-Methyl quercetin (**7**). LC-ESI-MS *m*/*z* 317 [M + H]^+^, UV *λ*_max⁡_ = 255, 367.
 5-*O*-Methyl aromadendrin (**8**). LC-ESI-MS *m*/*z* 303 [M + H]^+^, UV *λ*_max⁡_ = 290.
 5-*O*-Methyl kaempferol (**9**). LC-ESI-MS *m*/*z* 301 [M + H]^+^, UV *λ*_max⁡_ = 269, 365.

### 2.5. Acid Hydrolysis of ** 1**

Acid hydrolysis was performed using 1% HCl in MeOH at 100°C for 2 h from 9.3 mg of **1**. For aglycone detection, the final aqueous acidic mixture was extracted with EtOAc; then the aqueous layer was neutralized for determination of the released sugar moieties using silica gel plates with CHCl_3_ : MeOH (6 : 4). The anisaldehyde reagent was employed as spray for detection of the galactose from hydrolised sample which showed the same greenish gray pattern used galactose.

### 2.6. Determination of Total Phenolic Content

The total soluble phenolic content of the EtOH extract, hexane, EtOAc, and MeOH:H_2_O fractions (1 mg/mL) was determined with the Folin-Ciocalteu reagent according to the method of Slinkard and Singleton [14] with modification using gallic acid as a standard phenolic compound.

### 2.7. DPPH^∙^ Radical Scavenging Assay, ABTS^+∙^ Radical Cation Decolorization Assay, and Antioxidant Activity in Linoleic Acid Oxidation

The free radical scavenger activity DPPH [3], the radical cation decolorization assay ABTS [15], and the antioxidant activity in linoleic acid oxidation [16] of EtOH, hexane, AcOEt, MeOH:H_2_O extract, and fractions were determined.

### 2.8. Statistical Analysis

All samples were analyzed in triplicate unless stated otherwise, and the results were expressed as the mean ± standard deviation. All statistical analyses were carried out using the Microsoft Excel software package (Microsoft Corp., Redmond, WA, USA). 

## 3. Results and Discussion

The ethyl acetate fraction (EtOAc) from jandaíra geopropolis was chromatographed over Sephadex LH-20, and reverse-phase HPLC yielded compounds **1**–**9**. The structures of **1**–**8** were established by analysis of the spectral data, including 2D NMR and LC-ESI-MS. The APT NMR spectra of compound (**1**) (C_15_H_18_O_8_, LC-ESI-MS *m/z *326 [M + H]^+^) showed duplicated sugar carbon signal patterns. The dual peaks in the ^1^H NMR spectra of compound (**1**) indicated the presence of both *α*- and *β*-anomers, along with two doublets for the aromatic hydrogen system AA′BB′ at *δ* 7.5 (H-2, 6 *d*, *J* = 8.7 Hz) and *δ* 6.7 ppm (H-3, 5, *d*, *J* = 8.7 Hz) and two doublets showing the existence of *trans-*olefin systems at *δ* 7.7 (H-7, *J* = 15.9 Hz) and *δ* 6.4 ppm (H-8, *J* = 15.9 Hz), thus indicating the presence of the (*E*)-*p*-coumaroyl portion. Two anomeric protons were found in a 2 : 1 ratio at *δ* 4.9 (H-1*α*, *d*, *J* = 3.3) and *δ* 4.3 ppm (H-1*β*, *d*, *J* = 7.8), with the signals related to sugar protons between *δ* 4.4 and *δ* 2.9 ppm. The APT spectrum showed signs of a 1,4-disubstituted benzene pattern with the peaks from methine carbons at *δ* 130.4 (C-2,6) and *δ* 115.8 ppm (C-3,5), an oxygenated carbon at *δ* 159.9 (C-4), another quaternary carbon at *δ* 125.1 ppm (C-1), a carbon ester at *δ* 168.7 (C-9), and two olefinic carbons at *δ* 144.9 (C-7) and *δ* 113.7 ppm (C-8) relative to the *trans* coumaroyl portion. In addition to these signals, the APT spectrum also showed the presence of 11 signals to a hexose in different proportions. The values for the pyranose agree with galactose. The location of the phenylpropanoid group was concluded to be the C-6 position of the galactose moiety based on the evidence of the downfield shift of the C-6 methylene signals in the ^1^H NMR and APT NMR spectra. The HMBC spectrum showed a correlation of the methylene hydrogens of galactose to *δ* 4.4 (H-6*α*) and *δ* 4.1 ppm (H-6*β*) with the carbonyl group in *δ* 166.9 showing the ester linkage in the 6′ position of the sugar. The complete structure assignment of 6-*O*-*p*-coumaroyl-*D*-galactopyranose was performed via a detailed analysis of the COSY, HSQC, and HMBC spectra (Table 1). The LC-ESI-MS spectrum revealed the peak at *m/z* 326.9 [M + H]^+^ due to the molecular ion, at *m/z* 309 due to the loss of water, and at *m/z* 165 due to the loss of galactose. The peak at *m/z* 147 refers to the loss of two molecules of water and galactose. 

**Figure 1:**
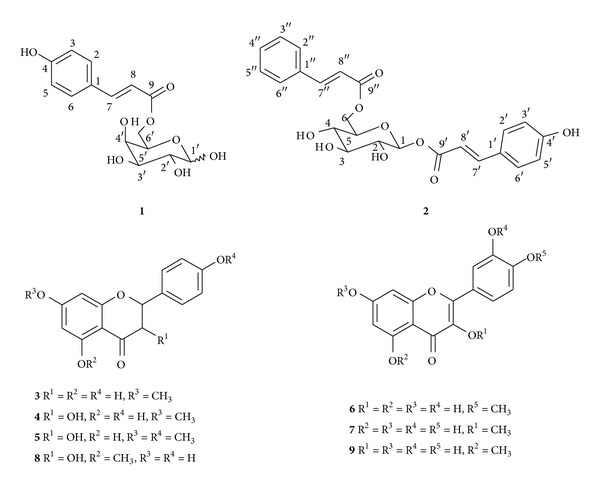
Structures of phenolic acids and flavonoids found in geopropolis.

The acid hydrolysis of (**1**) yielded coumaric acid and *D*-galactose. The phenylpropanoid group was concluded to be the C-6 position of the galactose moiety from the evidence of the downfield shift of the C-6 methylene signals in the ^1^H and ^13^C NMR spectra. Therefore, we assigned the structure of compound (**1**) as 6-*O*-*p*-coumaroyl-*D*-galactopyranose. This identification of 6-*O*-*p*-coumaroyl-*D*-galactopyranose (**1**) is first reported in the literature. The related derivative 6-*O*-*p*-coumaroyl-*D*-glucopyranose has been isolated from several plant species, such as *Prunus buergeriana* [17], *Flacourtia indica* [18], and *Petrorhagia velutina* [19]. The known compounds were identified as 6-*O*-cinnamoyl-1-*O*-*p*-coumaroyl-*β*-*D*-glucopyranose (**2**) [20], 7-*O*-methyl naringenin (**3**), 7-*O*-methyl aromadendrin (**4**) [21], 7,4′-di-*O*-methyl aromadendrin (**5**), 4′-*O*-methyl kaempferol (**6**), 3-*O*-methyl quercetin (**7**) [22], 5-*O*-methyl aromadendrin (**8**) [23], and 5-*O*-methyl kaempferol (**9**) [22], as confirmed by comparison of the spectroscopic data (UV, IR, MS, and NMR) with the corresponding data for the standards or literature values. The chemical structures of the isolated compounds are shown in Figure 1. This is the first report of compounds **1**–**9** from the geopropolis of *Melipona subnitida* (jandaíra) stingless bees.

 The amount of total phenolics was estimated using the Folin-Ciocalteu reagent, ranging from 63.9 ± 8.6 mgGAE/g (gallic acid equivalent per gram of extract) in the EtOH extract and 40.0 ± 7.8, 25.6 ± 0.5, and 115.8 ± 0.8 mgGAE/g in the MeOH:H_2_O, hexane, and EtOAc fractions, respectively. The highest total phenolic level was detected in the EtOAc fraction and the lowest in the hexane fraction. Three different methods were used to determine the antioxidant properties of the geopropolis, which allowed us to obtain information about the activity of these extracts during the different stages of the oxidation reaction [24]. The methods used included the inhibition of *β*-carotene, cooxidation in a linoleic acid model system, and DPPH and ABTS scavenging. The extracts and fractions assayed prevented the bleaching of *β*-carotene in the carotene/linoleic acid mixtures (Table 2). However, different fractions exhibited varying degrees of antioxidant capacity. The antioxidant activity was determined based on the inhibition of the coupled oxidation of *β*-carotene and linoleic acid at *t* = 60 min (Table 2). The antioxidant activity was 35.7 ± 4.0, 55.1 ± 1.9, 26.3 ± 3.5, and 20.7 ± 1.6% for the EtOH, EtOAc, hexane extracts, and the MeOH:H_2_O fraction, respectively. 

In conclusion, phenylpropanoids and flavonoids were isolated from the most active EtOAc fraction (Figure 1) that had not been tested because they had only been isolated in small amounts. The literature includes a report that shows that the flavonoid **7** (3-*O*-methyl quercetin) is active in DPPH radical scavenging and *β*-carotene-linoleic acid bleaching assays [25]. The antioxidant activity of the EtOAc fraction studied in this work may be related to the antioxidant abilities of the flavonoids and phenylpropanoids that were isolated. 

## Figures and Tables

**Table 1:** ^
1^H and ^13^C NMR (300/75 MHz, DMSO-*d*_6_) of compound (**1**).

Position	*δ*_*C*_	*δ*_*H*_	HMBC correlations
9	166.68	—	H-8, H-7
4	159.89	—	H-3,5; H-2,6
7	144.95	—	H-2,6
7	144.82	7.7 (*d*, *J* = 15.3)	—
2,6	130.40	7.5 (*d*, *J* = 8.7)	H-7
1	125.05	—	H-3,5; H-8
3,5	115.78	6.7 (*d*, *J* = 8.7)	—
8	113.97	6.4 (*d*, *J* = 15.9)	—
1′*α*	92.32	4.91 (*d*, *J* = 3.3)	2*α*, 5*α*
1′*β*	96.93	4.32 (*d*, *J* = 7.8)	2*β*
5′*β*	76.44	3.2 (*m*)	4*β*, H-3*β*
3′*β*	74.72	2.9 (*t*, *J* = 8.4)	4*β*, 1*β*, 5*β*
2′*β*	73.60	3.4 (*m*)	—
5′*α*	72.91	3.6 (*m*)	H-1′*α*
4′*β*	72.19	3.1 (*m*)	—
4′*α*	70.66	3.2 (*m*)	—
3*α*	70.24	3.1 (*t*, *J* = 8.4)	—
2′*α*	69.28	3.8 (*m*)	—
6′a	63.97	4.4 (*dd*, 12.0, 1.5)	—
6′b	63.97	4.1 (*d*, *J* = 11.1, 6.6)	4*β*

**Table 2:** Total phenolics and antioxidant activity of samples.

Extract or fraction	Total phenolic content (mgGAE/g)^a^	DPPH (EC_50_)^a^	ABTS (EC_50_)^a^	*β*-Carotene bleaching (% O.I.)^b^
EtOH	63.9 ± 8.6	27.0 ± 0.4	12.2 ± 0.1	35.7 ± 4.0
Hexane	25.6 ± 0.5	239.3 ± 1.1	32.3 ± 0.3	26.3 ± 3.5
EtOAc	115.8 ± 0.8	10.1 ± 0.0	4.3 ± 0.0	55.1 ± 1.9
MeOH : H_2_O	40.0 ± 7.8	38.5 ± 0.5	26.2 ± 0.3	20.7 ± 1.6
Ascorbic acid		2.1 ± 0.0	—	
Trolox		—	3.0 ± 0.1	81.3 ± 0.2

Mean value ± standard deviation: *n* = 3.

^a^Antioxidant concentration required to reduce the original radical population by 50%.

^b^Oxidation inhibition.
